# The Reversal of Roux en Y Gastric Bypass: Utilizing the Roux Limb in Response to Chronic Diarrhea in a Patient with Previous Multiple Bariatric Metabolic Surgeries

**DOI:** 10.1007/s11695-024-07242-0

**Published:** 2024-05-03

**Authors:** Mohamed Hany, Mohamed Ibrahim, Ahmed Zidan, Anwar Ashraf Abouelnasr, Bart Torensma

**Affiliations:** 1 Department of Surgery, Medical Research Institute, Alexandria University, 165 Horreya Avenue, Hadara, 21561 Alexandria Egypt; 2 Madina Women’s Hospital, Alexandria University, Hadara, Egypt; 3 Leiden University Medical Center (LUMC), Leiden, Netherlands

**Keywords:** Revision surgery, Roux en Y gastric bypass, Henley-Longmire interposition, Diarrhea, Internal herniation, Quality of life

## Introduction

Roux-en-Y gastri0063 bypass (RYGB), while efficacious for weight reduction, encompasses a spectrum of potential long-term adverse outcomes, encompassing both surgical and nonsurgical complications such as malnutrition, marginal ulceration, internal hernia, chronic diarrhea, and postprandial hypoglycemia. Among these, chronic diarrhea—characterized by the presence of three or more loose stools daily for a duration exceeding 4 weeks, coupled with a fecal weight surpassing 200 g per day—emerges as a significant postoperative concern [1].

This may stem directly from the surgical procedure or various treatable etiologies, each possessing distinct therapeutic interventions that facilitate restoring gastrointestinal function [2]. The incidence of diarrhea following RYGB is reported with considerable variability, with estimates ranging from 8 to 46.1%, as illustrated in studies by Potoczna et al. and El Labban et al., respectively [3, 4]. These variations hint at a potential association between the length of the common channel in RYGB and the prevalence of diarrhea. In severe instances, revisional surgery may become imperative [5].

In light of the reversal interventions, despite their effectiveness, they carry a heightened risk of complications and extended surgical times, especially in individuals who have undergone several bariatric metabolic surgeries (BMS) previously. Furthermore, while many surgical interventions for re-establishing intestinal continuity post-total gastrectomy exist, the jejunal Y-handle of Roux and the loop technique of Tomoda are predominantly employed. Conversely, the Henley-Longmire loop, utilized for intestinal transit restoration following partial gastrectomy, is seldom applied [6].

Another noteworthy complication post-RYGB is the development of chronic abdominal pain, one of the causes could be internal herniation (IH) that can lead to laparoscopic exploration. IH is a critical long-term challenge that may manifest after significant weight reduction [7]. Although prospective randomized trials advocate for the primary closure of the mesenteric defect during the initial surgery to diminish IH risk, its occurrence remains possible in patients lacking mesenteric defect closure [8, 9].

### Case Presentation

We demonstrate the feasibility of performing a laparoscopic reversal of RYGB in a 42-year-old female patient who had previously undergone multiple BMS revisions at the Medical Research Institute, Alexandria University, Egypt. This patient’s surgical history includes the placement of an adjustable laparoscopic band, followed by a laparoscopic sleeve gastrectomy (LSG), and ultimately, conversion to RYGB. She experienced significant diarrhea, steatorrhea, and chronic abdominal pain, which markedly impaired her quality of life.

This video demonstrates the laparoscopic restoration of normal food passage and the utilization of the Roux limb as a Henley-Longmire interposition between the gastric pouch and the remnant sleeve after the patient experienced diarrhea and steatorrhea, significantly impacting her quality of life.

## Results

The surgical procedure entailed laparoscopic exploration with adhesiolysis performed at the gastric pouch, followed by the measurement of intestinal limb lengths, which revealed an internal hernia within Petersen’s space. This hernia was subsequently reduced, and the lengths of the intestinal limbs were accurately measured. A segment of the Roux limb was interposed to facilitate the connection between the pouch and the sleeve remnant, derived from a previous reconstruction of RYGB. The remaining portion of the alimentary limb was then re-anastomosed to the biliary limb before the jejunojejunostomy. Intraoperative endoscopy was employed to confirm patency and conduct leak testing (Appendix Fig 1).

A drain was placed and subsequently removed on the second postoperative day. The operation lasted 100 min, with the patient being discharged after a 3-day hospital stay. A postoperative CT scan of the abdomen with oral contrast indicated normal dye passage. Notably, the patient experienced cessation of diarrhea and reported no episodes of abdominal pain during follow-up appointments at 1 and 3 months; we advised the patient to avoid exposure to the risk factors (non-steroid anti-inflammatory drugs, smoking, and alcohol use) and undertake a 6-month course of proton pump inhibitors.

## Conclusion

Diarrhea following RYGB poses a significant concern, impacting patient quality of life and potentially reducing the surgery’s benefits. Patient education on this complication and discussions on treatment and management strategies are essential. The Roux limb for interposition offers a safe and effective alternative to the higher risk gastro-gastrostomy, especially after multiple revisions. This method is a viable option when other treatments fail. Additionally, a thorough evaluation of intestinal limbs and correction of hernial defects during revisional surgery is vital to prevent further complications, ensuring optimal patient outcomes.

### Electronic supplementary material

Below is the link to the electronic supplementary material.

Supplementary file1 (MP4 386 MB)

## Figures and Tables

**Fig. 1:**
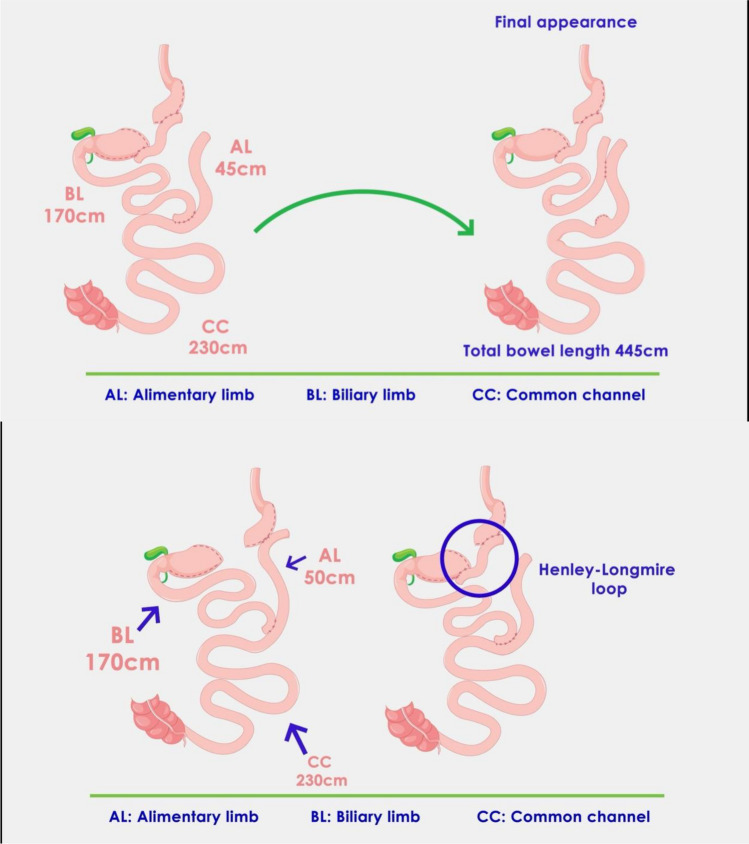
Schematic overview of the operation

## Data Availability

Data is available with the corresponding author.

## References

[1] Sollier C, Barsamian C, Bretault M (2020). Diagnostic and therapeutic management of post-gastric bypass chronic diarrhea: a systematic review. Obes Surg.

[2] Arman GA, Himpens J, Bolckmans R (2018). Medium-term outcomes after reversal of Roux-en-Y gastric bypass. Obes Surg.

[3] Potoczna N, Harfmann S, Steffen R (2008). Bowel habits after bariatric surgery. Obes Surg.

[4] El Labban S, Safadi B, Olabi A (2015). The effect of Roux-en-Y gastric bypass and sleeve gastrectomy surgery on dietary intake, food preferences, and gastrointestinal symptoms in post-surgical morbidly obese Lebanese subjects: a cross-sectional pilot study. Obes Surg.

[5] Ghiassi S, Higa K, Chang S (2018). Conversion of standard Roux-en-Y gastric bypass to distal bypass for weight loss failure and metabolic syndrome: 3-year follow-up and evolution of technique to reduce nutritional complications. Surg Obes Relat Dis.

[6] Longmire WPJ, Beal JM (1952). Construction of a substitute gastric reservoir following total gastrectomy. Ann Surg.

[7] Torensma B, Hany M, Bakker MJS (2022). Cross-sectional E-survey on the incidence of pre- and postoperative chronic pain in bariatric surgery. Obes Surg.

[8] Skidmore A, Aarts EO (2021). Preventing Peterson’s space hernia using a BIO synthetic mesh. BMC Surg.

[9] Kristensen SD, Gormsen J, Naver L (2021). Randomized clinical trial on closure versus non-closure of mesenteric defects during laparoscopic gastric bypass surgery. Br J Surg.

